# Development of an Elastic, Electrically Conductive Coating for TPU Filaments

**DOI:** 10.3390/ma14237158

**Published:** 2021-11-24

**Authors:** Henriette Grellmann, Mathis Bruns, Felix Michael Lohse, Iris Kruppke, Andreas Nocke, Chokri Cherif

**Affiliations:** Institute of Textile Machinery and High Performance Material Technology, Technische Universität Dresden, 01062 Dresden, Germany; mathis.bruns@tu-dresden.de (M.B.); felix_michael.lohse@tu-dresden.de (F.M.L.); iris.kruppke@tu-dresden.de (I.K.); andreas.nocke@tu-dresden.de (A.N.); chokri.cherif@tu-dresden.de (C.C.)

**Keywords:** TPU coating, electrically conductive filaments, electromechanical characterization, carbon nanotubes, smart textiles

## Abstract

Electrically conductive filaments are used in a wide variety of applications, for example, in smart textiles and soft robotics. Filaments that conduct electricity are required for the transmission of energy and information, but up until now, most electrically conductive fibers, filaments and wires offer low mechanical elongation. Therefore, they are not well suited for the implementation into elastomeric composites and textiles that are worn close to the human body and have to follow a wide range of movements. In order to overcome this issue, the presented study aims at the development of electrically conductive and elastic filaments based on a coating process suited for multifilament yarns made of thermoplastic polyurethane (TPU). The coating solution contains TPU, carbon nanotubes (CNT) and N-Methyl-2-pyrrolidone (NMP) with varied concentrations of solids and electrically conductive particles. After applying the coating to TPU multifilament yarns, the mechanical and electrical properties are analyzed. A special focus is given to the electromechanical behavior of the coated yarns under mechanical strain loading. It is determined that the electrical conductivity is maintained even at elongations of up to 100%.

## 1. Introduction

Since DuPont first introduced elastic polyurethane (PU) fibers under the brand name Lycra in 1962, a wide range of applications for yarns with high structural elongation has been developed [1]. In the clothing industry, elastic fibers are used to produce near-body clothing products, such as sportswear and swimwear [2,3]. In addition, the demand for elastic textiles is also increasing in the field of high-performance material technology with applications such as tailored medical compression [4], adaptive smart textiles [5] and novel fiber–elastomer composites [6]. Many of these novel fields of application require the storage and transmission of electrical energy. For example, many use cases of soft robotics or smart textiles can only be realized by using electrical sensors and actuators [7,8]. Sensors collect significant information on the intrinsic deformation and stress conditions present in the component, and textile actuators make it possible to respond to the collected information in an adequate manner.

For the transmission of electrical energy and information, electrically conductive fibers and filaments are required, whose mechanical stretchability are in the same order of magnitude as that of the textile base material and the matrix material. Conventional electrically conductive filaments, such as copper cables and carbon fibers, are limited in their applicability, due to their very low elasticity and high tendency to break at small bending radii [9]. Highly elastic filaments, on the other hand, are usually made of a polymeric base material and therefore have very low electrical conductivities. This publication aims to combine electrical conductivity and high mechanical stretchability in one fiber material.

Qin et al. identified three ways to produce highly elastic, electrically conductive fibers:

- Functionalizing elastic filaments with an electrically conductive coating,
- Achieving sufficiently structural elongation by tailored geometric arrangement of non-elongatable but electrically conductive yarns,
- A combination of both variants [9].

Coating elastic-polymeric-based filaments with an electrically conductive coating is a particular challenge. The main reason for this is that electrically conductive materials are usually stiff and brittle and therefore do not form a good bond with the textile base material. For example, Cao et al. have developed a multistep process to surround polyurethane filaments with a coating of silver nanowires. For this purpose, first a coating of sticky liquid PU is applied to the PU base fiber before a prepared film consisting of silver nanowires is rolled around the fiber [10]. Yang et al. and Zhang et al. developed similar processes for wrapping elastic filaments with carbon nanotube (CNT) sheets. For this purpose, the elastic filament is first coated with an electrolyte solution and then wrapped with a CNT sheet or a CNT/polyaniline sheet, respectively [11,12]. In these experiments, highly stretchable, electrically conductive fibrous structures could be developed. However, a major drawback lies in the complex multistep fabrication process, because, due to the coating by means of wrapped films of silver nanowires or CNT sheets, only short fiber sections can be functionalized to be electrically conductive, making mass production difficult to realize [10,11].

Wang et al. [13], on the other hand, coated highly elastic PU fibers with a CNT dispersion by using the intermediate step of twisting the PU filaments several times with cotton fibers. This resulted in a stable bond between the wrapped fiber and the coating, because the functional groups of the CNT formed hydrogen bonds to the hydroxyl groups of the cellulose [14]. The realized sensors can withstand elongations of up to 300%, which is only possible because the non-stretchable cotton fibers are applied in a spiral geometry around the elastic PU filaments by the twisting process [13]. This structural elongation is also exploited in other electrically conductive yarns that are expected to exhibit high stretchability. For example, PU yarns that are firmly bonded to copper wires in a pre-stretched state form a helical structure when they are relaxed [15]. Furthermore, electrically conductive yarns can be subjected to a structural elongation by means of knitting [16] or braiding [17], so that the resulting yarn structures can be used, for example, as strain sensors.

On the one hand, exploiting the structural elongation present in various yarn constructions is advantageous, as larger cross-sections of the electrically conductive yarn material can be used, and this improves the electrical conductivity. On the other hand, the diameter of the textile linear product increases considerably, and this is negative for numerous applications and processing variants. Furthermore, an additional processing step is necessary making production more time-consuming and cost-intensive.

In order to overcome this drawback and to introduce a novel method of producing electrically conductive and elastic fibers, this study presents a possibility to surround highly elastic yarns made of thermoplastic polyurethane (TPU) with an electrically conductive coating. In this way, TPU, a classic textile fiber product that is already used in many applications, can be equipped with additional functionality through a subsequent processing step. This opens up new fields of application in the area of smart textiles and soft robotics, where the electrically coated elastic yarns can be used as sensors and actuators.

## 2. Materials and Methods

### 2.1. Used Materials

TPUs are block copolymers consisting of hard and soft segments. The hard segments comprise a diisocyanate and a polyol, thus forming urethane groups (–NHCO–O–). The soft segments, on the other hand, consist of a polyester or polyether polyol. At the usage temperature, the soft segments are below their glass-transition point, but the hard segments determine the solid aggregate state and the mechanical strength of the material. Thus, the entropy–elastic soft segments cause the high elasticity of the polymer, whereas the semi-crystalline hard segments prevent the polymer chains from gliding off against each other [18]. When TPU is heated, the intermolecular bonds between the hard segments are broken, and the polymer becomes liquid, so that it can be melt-spun.

TPU is the basic material for many textile applications in the clothing industry, as well as in the development of customized high-performance materials. Equipping this versatile fiber material with an electrically conductive coating provides new possibilities for functional integration in various areas, such as smart textiles and soft robotics. The use of coating technology offers particular advantages, because a coating can generally be applied to different base yarns, as long as it is ensured that a long-term stable bond is created between the fiber and the coating. In addition, the coating can be applied in an additional process step after the spinning process, so that the primary spinning process is not affected. Furthermore, the contacting of conductive coatings is simpler than that of an electrically conductive fiber core surrounded by an insulating coating.

For the coating process, the polyether-based TPU grade Desmopan 9370A from Covestro AG (Leverkusen, Germany) [19] and the masterbatch Plasticyl TPU 1001 from Nanocyl SA (Sambreville, Belgium) [20] are used. Both polymers are based on TPU and offer, therefore, a low Young’s moduli and high elongation at beak. Plasticyl TPU 1001 contains multiwall CNT with an average diameter of 9.5 × 10^−9^ m and a length of 1.5 × 10^−6^ m. As the masterbatch is a compound of TPU and 10 wt% CNT, it offers a specific electrical resistivity of 361 Ωcm and can be used to develop an electrically conductive coating solution. In order to reduce the amount of CNT contained in the polymer, the masterbatch is blended with pure TPU Desmopan 9370A. Thereby, polymer compounds containing 2 to 10 wt% CNT were produced. These compounds, consisting of TPU and CNT, form the solid content of the coating solution.

### 2.2. Development of the Coating Solution and Coating Process

The TPU–CNT compounds were dissolved in N-Methyl-2-pyrrolidone (NMP) provided by VWR Chemicals (Radnor, PA, USA). Before the materials are dissolved, they are dried at a temperature of 80 °C for 24 h. For the dissolution, the TPU–CNT–NMP mixtures are left on a stirring plate, at room temperature, for 5 days, to obtain a homogeneous solution.

The coating was applied to mono-component TPU multifilament yarns made of Desmopan 9370A. The yarns were produced on a bicomponent melt-spinning plant of Dienes Apparatebau GmbH (Mühlheim am Main, Germany). These yarns consist of 60 filaments with a roving fineness of (53 ± 2) tex, an average elongation at break of (717 ± 85)% and a Young’s modulus of (37.0 ± 4.4) kPa [21].

In order to realize an electrically conductive coating for highly stretchable filaments, various specifications of the coating solution made of TPU, CNT and NMP were produced. On one hand, the solid content in the solution (TPU + CNT) was varied in the range from 2 to 10 wt%, while the CNT content in the solid content was kept constant at 10 wt%. On the other hand, the solid content was kept constant at 6 wt%, while the CNT content in the solid content was varied in the range from 2 to 10 wt%.

Prior to the coating process, the TPU filament yarns were cut to a length of 150 mm and were loaded with weights of 20 g. For each specification of the coating solutions, 14 rovings were coated at room temperature. For this purpose, the weighted yarns were completely dipped into the coating solution for a short time. Immediately after coating, coagulation was performed in a water bath for 60 s at 50 °C. This was absolutely necessary, since the solvent NMP must be removed from the coating solution as quickly as possible. If this is not performed, the NMP can penetrate into the filaments and dissolve them, which results in a significant reduction of the mechanical properties. After coagulation, the coated yarns were dried for 30 min, at room temperature, with sufficient ventilation, before the coating and coagulation processes were repeated two more times. This increases the applied coating layer and reduces the electrical resistivity, without significantly reducing the mechanical strength of the coated yarn.

### 2.3. Test Methods

To determine the viscosity of the coating solutions, rheometric measurements were performed on a Haake RheoWin/Thermo Scientific Mars II from Thermo Fisher Scientific Inc. (Waltham, MA, USA). The measurements were carried out at room temperature.

A determination of the fineness in accordance with DIN EN ISO 2060 was not possible, due to the small length of the coated samples. Instead, the coating masses were determined by measuring the masses of the rovings before and after coating with a precision scale R200D by Sartorius (Göttingen, Germany). The difference in mass was determined for each specification on 14 samples.

Tensile tests were performed on a Zwicki Junior from ZwickRoell GmbH & Co. KG (Ulm, Germany) with a clamping length of 62.5 mm and a testing speed of 200 mm/min. Both the tensile tests up to break and the cyclic tensile tests were performed on 5 specimens for each specification. The cyclic tensile tests were carried out with a combined measurement of the electrical resistivity in order to determine the electromechanical behavior of the coated samples. Fifteen cycles were performed between 20% and 100% elongation.

Measurements of electrical resistivity were performed with a multimeter Keithley DAQ6510-7700 from Keithley Instruments Corp. (Solon, OH, USA) by using a four-wire method. Static electrical resistivity was measured on filament sections with a length of 100 mm. Of each coating specification, 14 samples were tested. The value for the electrical resistivity was determined after a waiting period of 60 s for each measurement, since a certain settling behavior can be observed after closing the electric circuit. The determinations of the electrical resistivity during cyclic tensile tests were also carried out with a multimeter Keithley DAQ6510-7700 (Keithley Instruments Corp., Solon, OH, USA), but, according to the clamping length, only filament sections of 50 mm in length were measured.

## 3. Results

**Figure 1:**
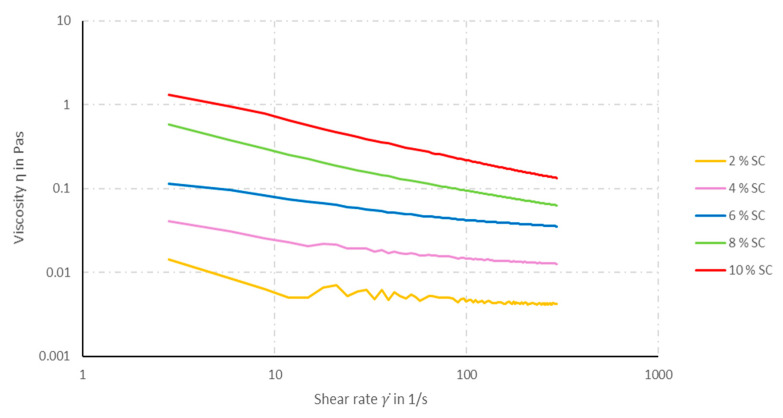
Viscosity of the coating solution as function of the SC with a constant CNT content of 10 wt% in the SC.

**Figure 2:**
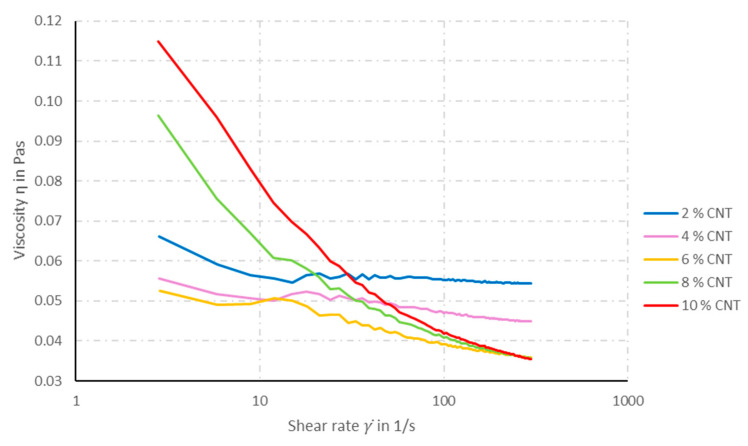
Viscosity of the coating solution as function of the CNT content in the SC; SC is kept constant at 6 wt%.

The solid content (SC) in the coating solution is composed of TPU and CNT. If the amount of CNT in the SC is kept constant at 10 wt%, the viscosity of the solution increases with increasing SC (see Figure 1). At a constant SC, the solution’s viscosity increases with increasing CNT content in the SC (see Figure 2).

**Figure 3:**
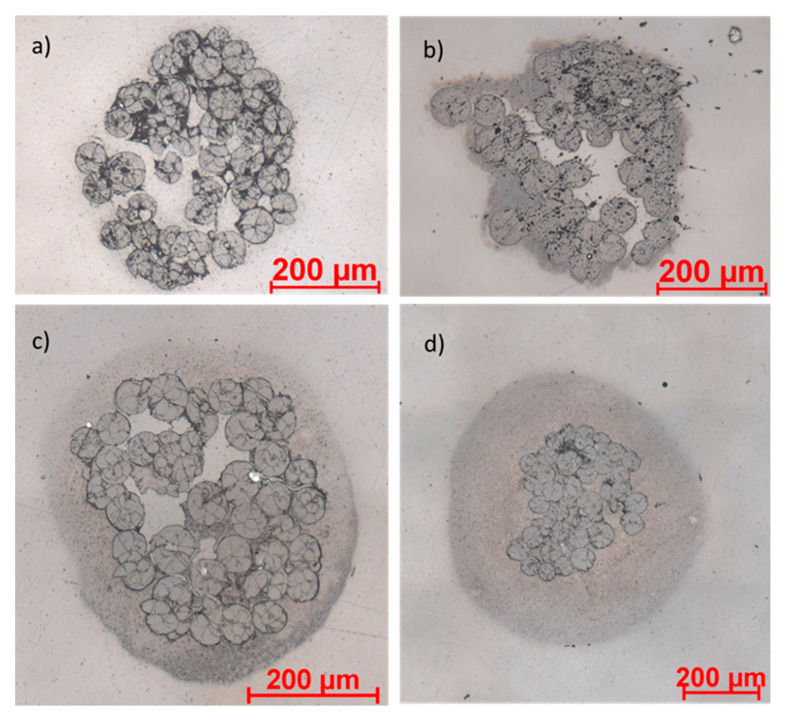
Cross-sections of coated multifilament yarns with 10 wt% CNT in the SC: (**a**) 2% SC, (**b**) 6% SC, (**c**) 8% SC and (**d**) 10% SC.

In all coating solutions, a shear-thinning rheological behavior can be observed. However, the decrease in viscosity by increasing shear rate becomes more pronounced the with increasing CNT content in the solution. Solutions with more than 10 wt% SC cannot be prepared, since the solids cannot be dissolved completely and, thus, no homogeneous coating solution can be obtained. The higher the viscosity of the solution, the greater are the coating volume and layer that remain on the multifilament yarn in each coating step. Figure 3 shows microscopy pictures of the cross-sections of TPU multifilament yarns coated with solutions containing different SCs.

It is clearly visible that an increasing SC does not only increase the applied coating amount but also leads to a compression in the yarn structure. This can be explained by the higher external pressure applied to the filaments by the greater amount of coating. This external pressure causes the filaments to rearrange themselves and thereby reduce the free volumes in between. Furthermore, it can be clearly seen that the thickness of the applied coating layer increases significantly with the increasing SC.

Table 1 shows the applied coating amount, the electrical resistivity and the mechanical properties of the coated yarns. Due to the multifilament structure of the yarn to be coated, it is not possible to determine the specific electrical resistivity of the applied coating, since the coating volume cannot be determined with high repeatability. Therefore, the electrical resistivity of the coated yarns was related to yarn length and coating amount, instead of volume.

The lowest electrical resistivity is measured for a coating solution containing 10 wt% solid content and 10 wt% CNT in the solid content. The application of this coating solution increases the Young’s modulus of the TPU multifilament yarn by one order of magnitude, while the elongation at break is reduced by half. Thus, the coated yarns exhibit lower elasticities than the uncoated yarns, but even the Young’s moduli of the coated yarns are well below 1 MPa, making them well suited for applications requiring a high degree of elasticity.

**Figure 4:**
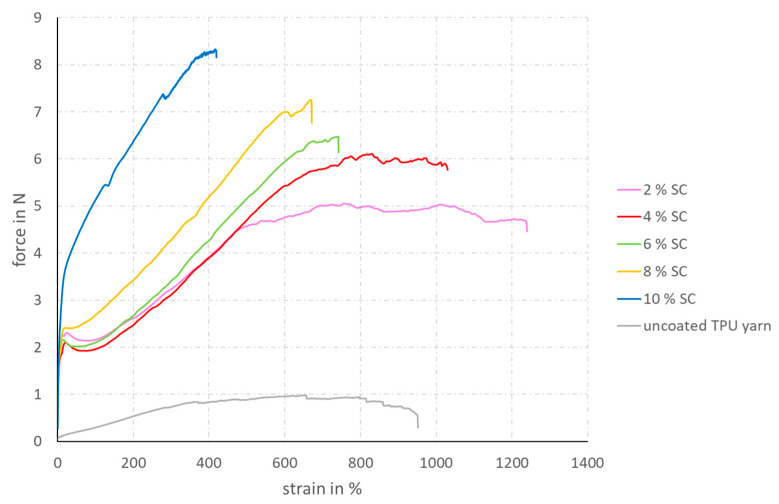
Strain–force diagram of TPU multifilament yarns coated with coating solutions containing different SCs and a constant content of 10 wt% CNT in the SC.

**Figure 5:**
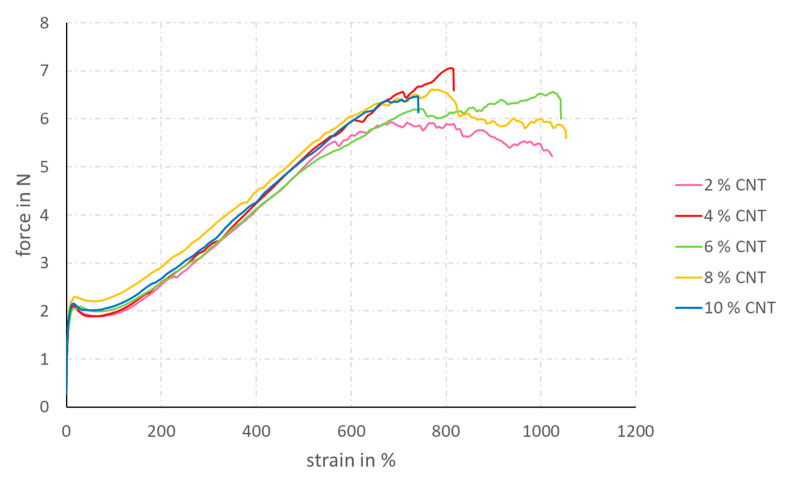
Strain–force diagram of TPU multifilament yarns coated with coating solutions containing a constant SC of 6 wt% and different CNT contents in the SC.

The stress–strain diagrams (see Figure 4 and Figure 5) clearly show that the application of the coating significantly increases the tensile strength of the filament yarns. As the coating creates a strong bond between the individual filaments, greater forces can be transmitted. In some coating solutions, which have a low solid content, even the elongation at break of the uncoated filaments can be exceeded, due to the improved filament cohesion. For the two coating solutions with 8 and 10 wt% solids content, the elongation at break decreases and the maximum tensile strength increases. All coating specifications containing less than 10 wt% SC show a decrease in force after a first peak at very low strain levels. This is a typical behavior of semi-crystalline polymers and thermoplastics. At a CNT content of 10 wt%, the brittleness of the coating, induced by the high amount of CNT, is so great that this point of relaxation does not occur anymore.

The presented coating process enables the production of electrically conductive and elastic filament yarns in a potential continuous automatable manufacturing process. Other methods are based on the wrapping of elastic filaments, either with CNT sheets [11,12], silver nanowires [10] or copper wires [15]. Even though these production methods show good mechanical and electrical properties, they require a multistep production process, because the stiff and brittle materials surrounding the filaments cannot form a cohesive bond with the textile base material and therefore need an intermedia step in order to enable a stable yarn compound. The coating solution presented in this study, however, is based on TPU itself and can therefore build up a cohesive bond with this versatile and often-used textile base material.

**Figure 6:**
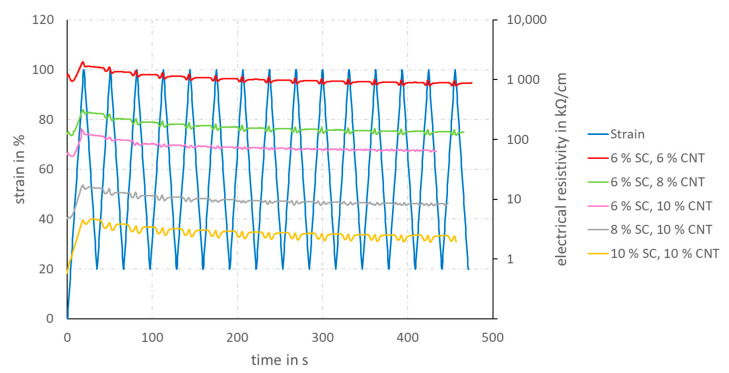
Cyclic-strain diagram with combined measurements of the electrical resistivity of coated TPU yarns; the light blue plot shows the mechanical strain depending on time, whereas the other plots describe the electrical resistivity, depending on time.

The coated yarns can be implemented in smart textiles and soft robotics for data and energy transmission. For reliable service, it is important that the electrical resistivity of the yarns is as constant as possible, even under mechanical strain loading. In order to measure the change in resistivity during elongation, cyclic tensile strain tests with combined resistivity measurement were carried out. All coated-yarn specifications whose electrical resistivity was within the measurement range of the static tests were analyzed. Figure 6 shows the correlation between strain, force and electrical resistivity for all electrically conductive yarn specifications.

It can be clearly observed that the electrical resistivity decreases with increasing solid and CNT concentration in the coating solution. The coating solution with 10 wt% SC and 10 wt% CNT in the SC exhibits the lowest electrical resistivity. For all coating specifications, it can be seen that the electrical resistivity increases at the first strain cycle, decreases slightly in further strain cycles and finally settles at an approximately constant level. Furthermore, it can be observed that the electrical resistivity reaches a significant peak at the maximum elongation, while it decreases when the yarn is relaxed. This can be explained by percolation theory. The CNTs build electrically conductive pathways in the polymer matrix. If mechanical loading is applied to the TPU–CNT compound, the CNT are pulled away from each other. Thereby, the conductive paths are interrupted, and the electrical resistivity rises, reaching its maximum at the maximum elongation. If the coated filament is relaxed again, the CNTs rearrange within the polymeric matrix and form new paths again, resulting in a lower resistivity.

**Figure 7:**
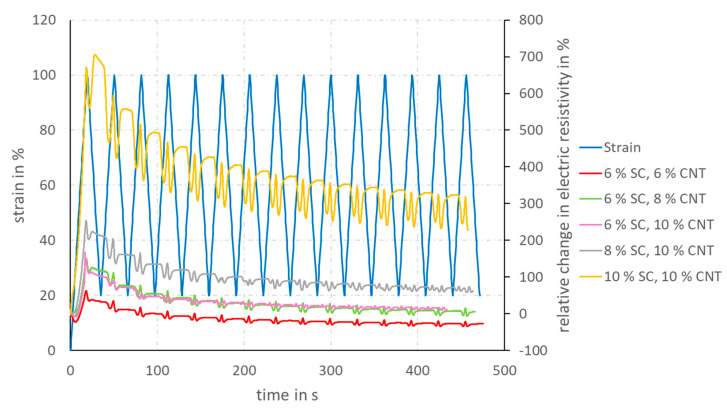
Cyclic-strain diagram of coated TPU yarns, showing the relative change in electric resistivity, depending on the strain; the light blue plot shows the mechanical strain, depending on time, whereas the other plots describe the electrical resistivity, depending on time.

No unambiguous relationship between elongation and electrical resistivity can be established for any specification. This behavior becomes even more apparent when the relative change in electrical resistivity is considered instead of the absolute electrical resistivity (see Figure 7).

The change in electrical resistivity is by far the greatest for the coating solution containing 10 wt% SC and 10 wt% CNT in the SC. For this specification, the electrical resistivity after several elongation cycles ranges from 220 to 330% relative to the initial resistivity. For specifications with lower SC (6% SC, 6% CNT; 6% SC, 8% CNT; 6% SC and 10% CNT), on the other hand, the electrical resistivity reaches the same order of magnitude as the initial resistivity after several elongation cycles. For the coating specification with 6 wt% SC and 6 wt% CNT in the solid content, the electrical resistivity already drops after three elongation cycles to values that are below the initial resistivity, so that negative values are recorded for the change in electrical resistivity.

The lower the CNT content is in the coating solution, the lower is the change in electrical resistivity in the first strain cycle. As a result of the mechanical stress, which moves the electrically conductive particles in longitudinal direction away from each other, the resistivity increases. At the same time, transverse contraction occurs. The radius of the coated rovings decreases, while the length increases. It can be assumed that the compression in radial direction increases the packing density of the CNT, resulting in the formation of new electrically conductive paths. The strain load, on the other hand, leads to the disruption of the conductive paths. This interaction of viscoelasticity and compression of the conductive network as a result of transverse contraction leads to a complex, non-monotonic response of the resistivity signal as a function of strain [22]. In the first strain cycle, the separation of the conductive paths due to strain and the increase of the CNT packing density due to transverse contraction are superposed.

At the same elongation, the transverse contraction is relatively greater for smaller volumes than for specimens with a large volume. The more SC is contained in the coating solution, the greater is the applied coating thickness and, thus, the electrically conductive volume. This means that the minimization of electrical resistivity caused by transverse contraction increases with decreasing SC in the solution. Therefore, the multifilament yarns that have been coated with solutions containing a small amount of solids show a lower increase in electrical resistivity during the first elongation.

After the first elongation, reorientation processes of the CNTs probably take place within the elastomeric matrix, so that more interconnections between the CNT are formed. Therefore, both the absolute electrical resistivity and the change in resistivity decrease. Nevertheless, secondary peaks or shoulder phenomena can be seen in each cycle. These are also due to a superposition of the resistivity change resulting from the strain load and the viscoelasticity respectively transverse contraction [22]. At this point, more in-depth experimental and theoretical work would be required to understand and quantify the influences of transverse contraction and viscoelasticity in more detail in order to gain a deeper understanding of the material behavior of the CNT-filled TPU coating.

## 4. Conclusions

By applying a coating solution made of NMP, TPU and CNT, we equipped highly elastic yarns with electrical conductivity, without causing them to lose their elasticity. A coating solution containing 10 wt% SC and 10 wt% CNT in the SC shows an electrical resistivity of 1.5 ± 0.3 kΩ/cm. A multifilament yarn containing 60 filaments made of TPU that was coated with this solution reached an elongation at break of 387 ± 63%. Coating solutions containing less SC and less CNT reach even higher elongation at break, but they also show higher electrical resistivity. As the coating is TPU-based, it can form a cohesive bond with TPU filaments and thus offers high bonding strength. However, it is also imperative that coagulation is carried out after coating, so that the solvent contained in the coating cannot penetrate into the textile base filament and destroy its mechanical properties. Coagulation can be carried out in water at 50 °C.

The coating based on TPU and CNT offers great potential for incorporating electrically conductive yarns into highly elastic textiles. Application of the coating is neither time-consuming nor cost-intensive and can be carried out on different specifications of TPU yarns, making the coating very versatile. It can find use in soft robotics, fiber–elastomer composites or smart textiles for transmitting electrical current. A significant advantage of the coated yarns is the fact that the electrical conductivity changes only slightly under elongation stress and that reliable transmission of energy and information is thus ensured even under heavy loads.

## Figures and Tables

**Table 1:** Electrical and mechanical properties of multifilament yarns coated with different specifications of TPU–CNT–NMP solutions.

Specification	Coating Amount in mg/cm	Electrical Resistivity in kΩ/cm	Young’s Modulus in kPa	Elongation at Break in%
uncoated TPU yarn	0	>10^5^	37 ± 4	717 ± 85
2% SC, 10% CNT	0.9 ± 0.1	>10^5^	402 ± 16	780 ± 255
4% SC, 10% CNT	1.1 ± 0.1	>10^5^	381 ± 21	907 ± 77
6% SC, 10% CNT	1.5 ± 0.1	168.4 ± 68.2	357 ± 37	715 ± 31
8% SC, 10% CNT	2.5 ± 0.3	11.4 ± 2.6	384 ± 24	653 ± 48
10% SC, 10% CNT	7.7 ± 1.9	1.5 ± 0.3	467 ± 29	387 ± 63
6% SC, 2% CNT	1.6 ± 0.2	>10^5^	388 ± 23	729 ± 51
6% SC, 4% CNT	1.2 ± 0.1	>10^5^	344 ± 58	813 ± 103
6% SC, 6% CNT	1.7 ± 0.1	454.7 ± 137.6	372 ± 32	756 ± 75
6% SC, 8% CNT	1.6 ± 0.1	4.9 ± 2.8	397 ± 48	829 ± 141
